# Circular Labor Migration and HIV in India: Exploring Heterogeneity in Bridge Populations Connecting Areas of High and Low HIV Infection Prevalence

**DOI:** 10.1093/infdis/jiu432

**Published:** 2014-12-01

**Authors:** Tanvi Rai, Helen S. Lambert, Annick B. Borquez, Niranjan Saggurti, Bidhubhushan Mahapatra, Helen Ward

**Affiliations:** 1 Department of Infectious Disease Epidemiology, School of Public Health, Imperial College London; 2 School of Social and Community Medicine, University of Bristol, United Kingdom; 3 Population Council of India, New Delhi

**Keywords:** geographical connectedness, transients and migrants, HIV infections/epidemiology, India, risk factors, sexual behavior

## Abstract

***Background.*** The emerging human immunodeficiency virus (HIV) epidemics in rural areas of India are hypothesized to be linked to circular migrants who are introducing HIV from destination areas were the prevalence of HIV infection is higher. We explore the heterogeneity in potential roles of circular migrants in driving an HIV epidemic in a rural area in north India and examine the characteristics of the “sustaining bridge population”, which comprises individuals at risk of HIV acquisition at destination and of HIV transmission into networks at origin capable of sustaining an epidemic.

***Methods.*** Results of a behavioral survey of 639 male migrants from Azamgarh district, India, were analyzed using χ^2^ tests and logistic regression.

***Results.*** We estimated the size of various subgroups defined by specific sexual behaviors across different locations and over time. Only 20% fit our definition of a sustaining bridge population, with the majority making no apparent contribution to geographical connectedness between high- and low-prevalence areas. However, we found evidence of sexual contacts at origin that could potentially sustain an epidemic once HIV is introduced. Variables associated with sustaining bridge population membership were self-perceived HIV risk, current migrant status, and age.

***Conclusions.*** Circular migrants represent a heterogeneous population in terms of their role as a bridge group. Self-perception of heightened risk could be exploited in designing prevention programs.

India has a concentrated human immunodeficiency virus (HIV) epidemic, with an overall HIV infection prevalence of 0.31% [1]. The subepidemics across the country remain asynchronous between urban and rural areas, with a much higher prevalence in the former. However, the distribution so far, in which only 6 states were considered as having a high prevalence, is shifting, with a decrease in HIV infection in these high-prevalence states and an emergence of hotspots of increasing prevalence within low-prevalence states [1]. This pattern has been attributed to long-distance male migrant workers who circulate for paid employment between their native, largely rural locations with a low HIV prevalence to urban destinations with a higher HIV prevalence [1–3].

There is a significant body of evidence for the association between seasonal or circular labor workforce migration and HIV risk [4–9]. As a consequence, migrant workers have been viewed epidemiologically as a potential bridge population [10, 11], defined as a population transmitting infection from a high-prevalence group to individuals who would otherwise be at low risk of infection [12].

There are a number of ways in which migration may contribute to the spread of HIV infection, and this is dependent on the number of migrants, their sexual behavior at destination and origin, the sexual behavior of non-migrating members of migrant-sending communities, the HIV prevalence among sex partners of migrants at both locations, and the frequency and duration of migration episodes [10]. On an individual level, migration increases the risk of HIV acquisition for migrants by exposing them to potential sex partners who are drawn from communities with a higher HIV prevalence than that in the migrants’ native communities; migration may also lead to an increase in levels of sexual risk behaviors among migrants [13]. Sex while at the migrant destination could occur in response to a desire to have new sexual experiences, as a result of loneliness, material need, or coercion. Migration may interfere with the formation of stable relationships, leading to more casual and potentially riskier sexual encounters both at destination and origin [5, 6, 8, 14]. By depleting the number of men in migrant-sending communities, migration also influences patterns of sexual behavior within the nonmigrating sections of the community [13, 15, 16]. For example, female sex partners of migrant men may form new partnerships with nonmigrating men to cope with loneliness or for financial reasons [17, 18]. At the population level, 2 main hypotheses have been proposed to explain how migrants may be contributing to increasing prevalence in their areas of origin. These mirror the individual-level factors mentioned above. First, migration increases geographical connectedness between high- and low-prevalence areas; second, social disruption accompanying migration is associated with behavior changes in migrants and in nonmigrants who are left behind, such that networks supporting transmission are formed [19, 20].

Geographical connectedness is believed to be most important during the early phase of the epidemic in migrants' native villages [13]. Migration, and especially circular migration, in which migrants make repeated trips back to their homes, allows for an almost continuous connection between ≥2 locations with asynchronous epidemics [20]. During the early phase, this helps feed the epidemic in low-prevalence areas, but with time, as the epidemic becomes more established, the role of migration decreases [21]. However, bridging can only result in epidemic establishment and sustainment if infection is introduced into a network that supports further transmission, otherwise transmission meets a dead end. Studies examining the national epidemiology of HIV and risk profiles in India report that married women are largely sexually monogamous and that their risk of HIV acquisition is principally via their husbands [22–24]. Thus, in areas of low HIV prevalence, the group of migrants most likely contributing to the introduction and establishment of HIV are those who are connected to an open sexual network within communities at both ends of their migration circuit and who are thus at increased risk of HIV acquisition and transmission into a network that has the potential to sustain an epidemic at origin.

A cross-sectional study in 2 districts of high out-migration in India found that migrants reported a greater number of risky sexual behaviors than nonmigrants at both origin and destination [25] and therefore represented an interesting sample in which to investigate the heterogeneity in the potential role of migration in HIV transmission. In this article, we use data from one of those districts, Azamgarh, in Uttar Pradesh state, to estimate the relative size of each migrant subgroup according to epidemiological definitions of risk behavior. We then describe characteristics of the subgroup that is most likely to influence epidemic growth in this low-prevalence rural setting.

## METHODS

We performed a secondary analysis of data from a behavioral survey performed by the Population Council in 2008. Ethical clearance for the original study was received from the institutional review board of the Population Council. We used data from 1 site, Azamgarh District, in the northern state of Uttar Pradesh, an area of high out-migration where the prevalence of HIV infection is increasing among antenatal clinic attendees [26]. Detailed methods for conducting the behavioral survey have been reported elsewhere [25]. Briefly, the sample was recruited at the place of origin, when migrants were visiting their home villages. Men aged >18 years were recruited to include equal numbers of active migrants (ie, men who had migrated within the past 12 months) and return migrants (ie, men with a history of out-migration for work who had not migrated in the last 12 months). Active migrants' reported reasons for visits home included participation in seasonal agricultural work, vacation, attending marriages, and other events, and visits lasted an average of 5–6 months. These migration characteristics are consistent with rural-to-urban migration common in this part of North India, in which men travel without their families over long distances for several months per year to work in urban and peri-urban destinations. Such migrants remain as temporary, circular migrants rather than permanent migrants, keeping their families rooted in their home villages [27, 28].

We used this sample to define subgroups based on available data on sexual behavior, including timing (before or after migration), location of sex (at origin or destination), and type of sex partner (marital or non-marital). We defined casual sex as non-marital sex (ie, sex with any women outside of marriage); for unmarried participants, this would be equivalent to premarital sex. We defined a “traditional bridge population” as comprising men who reported having casual sex at destination and were either married or, if unmarried, who reported having sex at origin, as well. We defined a “sustaining bridge population” as comprising migrants who reported having casual sex at destination and also subsequently at origin during return visits. These men were therefore at increased risk of acquiring HIV at destination and transmitting it to an open sexual network at origin. Within this group, we also examined reported consistent condom use, to further quantify the group within the sustaining bridge population who were most likely to acquire HIV infection themselves and transmit it to their village-based open sexual network. This was based on men from within the sustaining bridge population reporting on their condom use with non-marital sex partners at destination and on subsequent trips to origin.

The characteristics of the sustaining bridge population were compared to those of the rest of the sample by using Pearson χ^2^ tests and odds ratios. All reported *P* values were 2 sided. Associations at a *P* value of < .2 were explored further by multivariate logistic regression. The final model included explanatory variables that were significant at the 5% level, and the associations were tested using likelihood ratio tests. The goodness-of-fit of the final model was tested using the Hosmer and Lemeshow χ^2^ test.

We also briefly examined the second hypothesis for the contribution of migration to increased prevalence in rural areas (behavior change) by quantifying the number of migrants who reported casual sex only after their first migration. We had no information on the sexual behavior of migrants' sex partners, which limited the investigation of this hypothesis.

## RESULTS

Data from 639 migrant men were available for this analysis, divided equally across active and returned migrants. The median age of respondents was 30 years. Returned migrants were significantly older than active migrants, with a 10-year difference in median age. Most respondents were currently married, with a greater percentage of married men among the returned migrants than the active migrants (97% vs 71%; *P* < .001).

**Figure 1.**
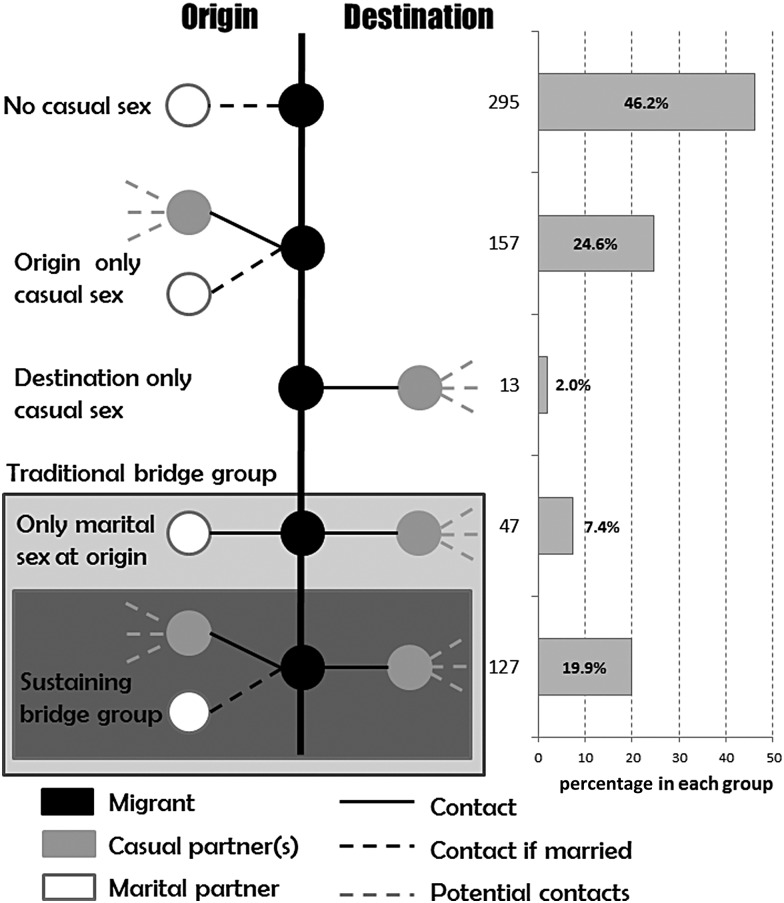
Distribution of migrant men in the sample according to their sexual behavior at origin and destination.

This distribution of migrants according to sexual behavior is shown in Figure 1. Overall, 174 (27%) formed a traditional bridge population, of which 127 (20%) formed the sustaining bridge population. Most of the others (295; 46% of the total sample) reported no casual sex at origin or destination, while the remaining groups reported casual sex but did not appear to constitute any kind of bridge population (Figure 1).

Among the sustaining bridge population (127 men), condoms were not used consistently by 66 men (52%) at destination, and nearly all these men (62 [93.9%]) did not use condoms consistently during casual sex at origin either. Thus, 9.7% of circular migrants in this analysis could be described as the “high-risk sustaining bridge population”.

Table 1 shows characteristics of the sustaining bridge population, compared with the rest of the sample. After control for potential confounding, 3 variables remained statistically significant: current age, migration status, and self-perceived risk of HIV infection. Self-perceived HIV infection risk was the strongest predictor, with men perceiving themselves to be at high to moderate risk being nearly 5 times as likely to belong to the sustaining bridge population, compared with men who believed their risk to be low. Younger men, although not the youngest group, were 2.5–4.5 times as likely to belong to the sustaining bridge population, compared with men aged >40 years, and returned migrants were more than twice as likely as active migrants to belong to the bridge population. Age confounded the effect of migration status on the outcome to some degree, although this appeared to be nonlinear. Among men aged 25–39 years, returned migrants were more likely than active migrants to form the sustaining bridge group, but this was not true for the oldest and youngest age groups.

**Table 1.** Univariate and Multivariate Logistic Regression Analysis Examining the Factors Associated With Sustaining Bridge Population Membership

Characteristic	Proportion (%)^a^	OR (95% CI)	Adjusted OR (95% CI)
Self-perceived HIV infection risk^b^			
High to moderate	38/82 (46.3)	4.446 (2.710–7.294)	4.803 (2.816–8.192)^c^
Low	81/498 (16.3)	1	1
Current age, y			
≤24	14/114 (12.3)	.900 (.407–1.990)	2.706 (.911–8.043)
25–29	41/163 (25.2)	2.160 (1.111–4.201)	4.530 (1.836–11.178)^c^
30–34	30/126 (23.8)	2.009 (1.001–4.031)	3.652 (1.557–8.568)^d^
35–39	28/131 (21.4)	1.748 (.867–3.523)	2.411 (1.027–5.662)^e^
≥40	14/104 (13.5)	1	1
Migration status			
Return migrant	73/319 (22.9)	1.462 (.988–2.164)	2.224 (1.282–3.858)^d^
Active migrant	54/320 (16.9)	1	1
Occupation			
Construction worker/miner/stonecutter/factory worker	42/199 (21.1)	2.229 (.642–7.742)	1.941 (.531–7.096)
Daily wage laborer/loader/petty trader	37/177 (20.9)	2.202 (.630–7.696)	1.725 (.465–6.397)
Driver/fisherman/other	22/71 (31.0)	3.741 (1.021–13.714)	3.646 (.933–14.241)
Contractor/artist/tailor/private salaried/government salaried	23/164 (14.0)	1.359 (.379–4.870)	1.219 (.324–4.585)
Agricultural worker	3/28 (10.7)	1	1
Have savings despite expenses			
Yes	114/544 (21.0)	1.672 (.899–3.110)	1.641 (.801–3.360)
No	13/95 (13.7)	1	1
Age at first migration, y			
≤19	88/399 (22.1)	1.451 (.956–2.201)	1.099 (.665–1.817)
≥20	39/239 (16.3)	1	1

A total of 127 men of 639 surveyed composed the sustaining bridge population.

Abbreviations: CI, confidence interval; HIV, human immunodeficiency virus; OR, odds ratio.

^a^ Data show the proportion of migrants belonging to the sustaining bridge population (n = 127) within the total surveyed migrant population (n = 639), according to the specified characteristics (%).

^b^ This question was not answered by all respondents.

^c^ *P* ≤ .001.

^d^ *P* ≤ .01.

^e^ *P* ≤ .05

Finally, we found little evidence of changes in sexual behavior occurring after migration, as most of the 290 men who reported casual sex following migration also reported it at origin before they first migrated, with only 62 (21.4%) reporting first casual sex during or after migration.

## DISCUSSION

In this analysis from Azamgarh in northern India, we have found considerable heterogeneity in the sexual behavior of migrants with respect to their potential roles in introducing and sustaining an HIV epidemic at origin. In this group of migrants, a minority (27%) formed any kind of sexual bridge population (ie, the traditional bridge population), with only 1 in 5 having the potential to link open sexual networks at destination and origin (ie, the sustaining bridge population). The biggest group comprised men who reported no casual sex at either location, while other men reported casual sex at one or the other location but not both. For most of the men reporting casual sex after they migrated, this was a continuation of premigration sexual behavior, which does not support the hypothesis that migration leads to increased risk taking.

This quantification of a sustaining bridge population, if reflected in other settings, has implications for models of transmission from areas of high HIV infection prevalence to low-prevalence areas. This subpopulation of men may be the most important for the introduction and sustainment of HIV in this low-prevalence setting because of their role in connecting potentially open sexual networks [13]. These men are at risk of both acquiring HIV in a high-prevalence area and introducing it to a sexual network supporting transmission at origin, which, as mentioned previously, is important in early stages of epidemic establishment. Once HIV is introduced, behaviors and networks at origin then become critical; casual sex at origin was reported by a significant proportion of migrants, and similar behaviors occur among nonmigrants. In the same study, >20% of nonmigrant men reported having sex with a non-marital partner in the last 12 months [25]. In addition, the behavior of wives of migrants is also important, as they may not form a transmission dead end but may, themselves, link into wider networks, as suggested in modeling and empirical studies from other contexts [13, 15, 17, 18]. New and innovative research methods would be required to examine sexual networks in these rural north Indian villages, to overcome the challenges posed by complex social structures and gender norms preventing disclosure of sexual behavior.

Men in the sustaining bridge group were aware of their increased risk. This matching of perceived and actual risk indicates that people identified themselves correctly and may encourage increased protective behavior [29–31]. Men aged 20–39 years and those who were no longer currently migrating were most likely to be in this group, suggesting cohort effects, with those who are older and have migrated for longer having had more time to accumulate the acquisition and transmission risks. Returned migrants may have had reduced exposure to HIV prevention messages because HIV campaigns are strongest in urban areas and migrant work sites, or it could be an artifact of the survey design, making it more difficult to compare ongoing risks at both origin and destination between active and returned migrants.

There are a number of limitations to this study. It is cross-sectional and thus does not allow inferences to be made about causality or the direction of relationship between associations. Sexual behaviors within the particular categories identified in this study may change over time, and the profiles appearing in one survey may be different if it is repeated after 5 years. The study covered periods that may have included several circular migrations, and there was no information on the number and duration of each of the migration episodes. Time is a key dimension in the definition and study of migration, especially when examining geographic connectedness. For example, in a mathematical modeling study, Coffee et al found that frequent return of migrants to origin was linked to higher incidence rates of HIV infection among their sex partners since they had more contacts with them, and, if the migrants were infected, these contacts were more likely to occur during the acute phase of infection [13]. On the other hand, increased frequency of return trips to origin could decrease the incentive to form sex partnerships at destination, reducing the role of migration in HIV spread [32]. The corollary to this, with a similar reduction in the role of migration in HIV spread, would be the case of very infrequent return trips, in which migrants may eventually cease to have any sex partnerships at origin. Sexual behavior was self-reported and subject to social desirability and recall biases. Additionally, there was no information on HIV status, and so it was not possible to investigate the association between reported risk behaviors and HIV infection. For instance, nonrandom migration with respect to infectious status is a possible explanation for an increased frequency of risky behavior among returned migrants, but it could not be tested here [33]. The sample was recruited from migrants who were available at origin villages to participate during the study period, and this may have introduced some further bias. Finally, the range of questions on the questionnaire and missing data corresponding to particular questions limited the potential explanatory variables that could be included in the analysis.

Despite these limitations, this study makes an important contribution toward a better conceptual understanding of how migrants affect HIV transmission. Similar to the studies from elsewhere, most of the studies from India have concentrated on sexual behavior at destination locations only [5, 34, 35]. Breaking down HIV risk according to acquisition and transmission risks and testing particular hypotheses helps to sharpen the focus on migrants as a risk group and allows for a more scientific interpretation of where risk lies. Characterizing heterogeneities in behavior among bridge and other key populations is crucial to estimating their contribution to the spread of HIV, as well as to designing appropriate interventions.

This study has important implications. In terms of policy, it demonstrates that eliding all circular migrants with HIV risk is simplistic and risks stigmatizing migrants. Further research should similarly aim to present a more nuanced understanding of risk that accounts for the heterogeneity within this population type. These findings also have implications for improvements in disease transmission models: data such as these, which identify the varying acquisition and transmission risks of migrants, could help to refine the identification of risk populations to more accurately predict the contribution of migrants as bridge populations in the spread of HIV. Finally, the finding that migrants in the sustaining bridge population are aware of their increased risk could be exploited in the design of migrant-focused HIV prevention programs.
